# Cases of Cancer Treated at the College Clinic by Prof. Brainard

**Published:** 1857-12

**Authors:** Edwin Powell

**Affiliations:** Late Assistant Surgeon of the U. S. Marine Hospital, Chicago


### ARTICLE III.

### CASES OF CANCER TREATED AT THE COLLEGE CLINIC BY PROF. BRAINARD.

1. CASE OF SCIRRHOUS CANCER OF PENIS—AMPUTATION—RECOVERY.
2. CASE OF EPITHILIAL CANCER OF FACE—EXCISION—GREAT AMELIORATION.
3. CASE OF ENCEPHALOID CANCER, SITUATED UPON THE SIDE OF THE ABDOMEN, OF LARGE SIZE—EXCISION—DEATH AFTER THREE WEEKS FROM HEMORRHAGE INTO THE BRONCHIAL TUBES.

REPORTED BY EDWIN POWELL, M. D., LATE ASSISTANT SURGEON OF THE U. S. MARINE HOSPITAL, CHICAGO.

Michael H——, aged 45, a laborer, a stout muscular man, and, excepting a slight sallowness, healthy looking, applied to the College Clinic, Oct. 17th, 1857.

*Present state.*—Over the base of the glans penis, and a little to the left side, was a large ulcer which perforated the prepuce. Its edges were sinuous, everted and raised, and overhanging a marked constriction which surrounded their base. The surface of the ulcer was covered over with firm sprouting granulations, and discharged offensive ichorous pus. The penis was enlarged to two or three times its natural size, and was indurated and hard. The prepuce was in a condition of congenital phymosis, and it, with the integument of the penis, was inflamed and swollen. The whole was extremely sore and tender to the touch. There was an enlargement of the glands of both groins.

*History.*—Two years previous he discovered a hard lump of the size of a pea in the left side of the glans penis, which enlarged for a period of six months, when it commenced to ulcerate; the ulceration gradually perforating the prepuce, and extending down the left side of the penis in circular form. During the time, he applied to several doctors, but received no benefit from treatment. He says cold water applied locally to the part relieved the itching and inflammation better than anything else.

The disease was pronounced to be cancerous, and accordingly amputation of the penis was proposed to the patient, to which he assented.

*Operation, Oct. 17th.*—The skin of the penis having been drawn forward, and the organ grasped with the left hand, the whole was severed with a single sweep of the knife within an inch of the pubis. The skin at once retracted, leaving the uncovered corpora cavernosa projecting prominently forward. Ligatures were applied, and the penis covered with wet lint. On examining the removed parts, the glans and corpora cavernosa, to within half an inch of the incision, were found to be infiltrated with the deposit of scirrhus. It is perhaps scarcely necessary to remark, that the object in dividing the skin as far back as possible was to prevent it, during the healing process, closing over the end of the stump, and occluding the orifice of the urethra. In amputations of an extremity, no result is more deprecated than a conical stump; in the case of the penis, however, that condition is most desirable, and the rules for the performance of the two operations are consequently reversed. The incision through the skin should in the latter be as far back as possible, while, as is well known, in the former it can scarcely be made too far forward.

*Nov. 5th.*—The healing process has progressed favorably, and is now nearly complete. The orifice of the urethra projects, and its lips are everted and pouting in the best possible manner. He has had no difficulty in voiding his urine, has been quite free from pain since the operation, and appears to be in good health. After the operation, the lactate of iron was prescribed for the patient in five grain doses three times a-day, which he continued to take for the space of four weeks. It had the effect of removing his former sallowness and increasing his appetite and strength. Discharged.

This case furnishes another instance of the association of cancer of the penis and congenital phymosis. Their very frequent coincidence has been noted by all who have recorded observations on the subject. Hey observed it in nine out of twelve examples of the disease which fell under his notice; a proportion very great, when we consider that the malformation is after all not a common one. Mr. Travers has thrown additional light upon the subject, by showing that Jews are seldom the subjects of cancer of penis. The importance and occasional difficulty of diagnosis between cancerous and venereal ulceration of the genitals will be generally admitted. To mistake the disease, on the one hand, would be a fatal error, and on the other an unpardonable mistake. The use of the microscope should not be omitted in doubtful cases. The examining of the discharge, and portions of the diseased mass, which may be removed for that purpose, together with the history of the case, should enable us in every instance to make a correct diagnosis.

2. CASE OF EPITHILIAL CANCER OF THE FACE—EXCISION—GREAT AMELIORATION.

John Harris, aged 55, a ship carpenter, presented himself at the College Clinic, Oct. 24th, with a disease presenting the following appearance:

A fungous growth occupies the whole of the left side of the face, extending from the eye to the lower border of the inferior maxillary bone, and from the nose to the ear behind, projecting three and a half inches from the face. The surface is of a dark brown color, produced by the application of wood ashes, which the patient has been in the habit of using.

There is a copious discharge of excessively fetid pus from the surface. The movements of the lower jaw are continually interfered with. The patient is emaciated, and has the sallow appearance peculiar to cancerous patients.

The history of the case as given by the patient is that some three years previous a discolored spot made its appearance in the center of the left cheek, which remained stationary for six months, when a small lump was developed in its center, which continued to increase in size uniformly for one year. At this time he consulted a *cancer* doctor, who applied a caustic plaster to the tumor, which caused the integument and a part of the tumor to slough. In a very short time after this a fungous growth commenced to spring up from the base, which has continued to grow quite rapidly until the present time.

During this period, the patient has been traveling about the country consulting different "*cancer doctors*," who each in turn promised him a cure and applied his plaster, but, instead of receiving any benefit, the disease seems to have been greatly aggravated.

TREATMENT.—The fungous growth was removed with a scalpel a little below the surrounding surface. The copious hemorrhage which followed was checked by the application of the actual cautery. Water dressings were applied to the part. No unpleasant symptoms followed the operation, the patient being able to walk to the office each morning the distance of one mile. The remaining portions of the tumor have since been removed from time to time by means of the knife and arsenical paste. After the entire removal of the tumor, the vapor of iodine was applied to the surface of the ulcer. The method of applying the vapor is—first, to place upon the ulcer a dressing of simple cerate spread upon lint, and next to place half a gr. of iodine folded in a bit of paper or cloth on the surface, over which you place a layer of oil silk and tinfoil. The dressing is to be kept in place by a roller. The heat of the part vaporizes the iodine; and the oil silk and tinfoil preventing its escape, it is applied equally and continuously over the surface of the ulcer. Under this treatment the surface commenced to cicatrize very rapidly, and at the end of three weeks it was only about one-fourth its original size. The fetid discharge is entirely absent. The patient's general health, however, did not improve with the local disease. He was affected with diarrhœa most of the time, but this was probably owing to the fact that he was unable to procure comfortable board and lodgings, on account of the offensiveness of his disease.

*Nov. 24th.*—The patient returned to his home in the country to-day. The original ulcer is no larger than a twenty-five cent piece and presents a healthy appearance. It will be observed that although the cure was not entirely complete at the time of the patient's going away, yet the improvement was very great.

The history of this case proves conclusively that if we are not able to cure advanced cases of cancerous disease, we can do very much towards relieving the local symptoms.

3 CASE OF ENCEPHALOID CANCER, SITUATED UPON THE SIDE OF THE ABDOMEN, OF LARGE SIZE — EXCISION — DEATH, AFTER THREE WEEKS, FROM HEMORRHAGE INTO THE BRONCHIAL TUBE.

James Owen, aged 30, an Irishman, of intemperate habits, applied to the College Clinic, Nov. 7th, 1857, with a tumor occupying the left lumbar and iliac regions of about five inches diameter. The skin covering the tumor was of the natural color, except on its most prominent part, at which point it seemed to be inflamed from friction of the clothes, and where it was traversed by veins, the enlargement of which was conspicuous. It was lobulated, and on a superficial examination seemed hard, but on a careful investigation a number of soft spots could be discovered. Some of these had been opened under the impression that they contained matter, but only dark blood was discharged. The patient has never suffered much pain in the part.

*History.*—Some two years previous a small kernel made its appearance under the integument of the abdomen, which has grown rapidly and uniformly since. The patient has, however, been able to follow his usual occupation, until within the last six months, when from its size and weight he was not able to labor.

TREATMENT.—The patient being brought fully under the influence of chloroform, two semilunar incisions, which joined each other at their extremities, were made over the center of the tumor. The two flaps thus formed were dissected back as speedily as possible, mostly with the finger, and the tumor was raised from its base. It involved all the parts down to the transversalis fascia. Ligatures were placed upon the bleeding vessels and the wound closed by sutures; a compress was placed over the wound, and a roller round the patient. In consequence of the enlargement of the vessels leading to the tumor, a considerable quantity of blood was lost during the operation, and the patient was a good deal depressed at its close. He was placed in bed, warmth applied externally, and stimulants administered internally.

At the end of a few hours the patient had rallied from the effects of the operation, and remained quite comfortable until the following day, when he was attacked with delirium tremens in its most violent form, which continued for three days, when it subsided under the influence of opium and stimulants.

From this time forward the patient progressed favorably until the twenty-first day after the operation, when he suddenly died with hemorrhage from the lungs. No autopsy could be obtained.

After the removal of the tumor, it was carefully examined with the microscope, and found to be a soft or encephaloid cancer. The wound had commenced to heal at the edges, and presented a healthy appearance. The cause of the patient's death was in nowise connected with the operation or wound.

